# Nitric Oxide/Nitric Oxide Synthase System in the Pathogenesis of Neurodegenerative Disorders—An Overview

**DOI:** 10.3390/antiox12030753

**Published:** 2023-03-20

**Authors:** Olga-Maria Iova, Gheorghe-Eduard Marin, Izabella Lazar, Ioana Stanescu, Gabriela Dogaru, Cristina Ariadna Nicula, Adriana Elena Bulboacă

**Affiliations:** 1 Faculty of Medicine, Iuliu Hatieganu University of Medicine and Pharmacy, 400349 Cluj-Napoca, Romania; 2 Department of Neurology, Iuliu Haţieganu University of Medicine and Pharmacy, 400012 Cluj-Napoca, Romania; ioanastane@yahoo.com; 3 Department of Physical Medicine and Rehabilitation, Iuliu Haţieganu University of Medicine and Pharmacy Cluj-Napoca, Viilor Street, No. 46-50, 400347 Cluj-Napoca, Romania; dogarugabrielaumf@gmail.com; 4 Department of Ophthalmology, Iuliu Hațieganu University of Medicine and Pharmacy, 400012 Cluj-Napoca, Romania; 5 Department of Pathophysiology, Iuliu Hațieganu University of Medicine and Pharmacy, 400012 Cluj-Napoca, Romania; adriana.bulboaca@umfcluj.ro

**Keywords:** nitric oxide, nitric oxide synthase, Alzheimer’s disease, Parkinson’s disease, amyotrophic lateral sclerosis, neurodegenerative disorders, superoxide dismutase 1

## Abstract

Nitric oxide, a ubiquitous molecule found throughout the natural world, is a key molecule implicated in many central and benefic molecular pathways and has a well-established role in the function of the central nervous system, as numerous studies have previously shown. Dysregulation of its metabolism, mainly the upregulation of nitric oxide production, has been proposed as a trigger and/or aggravator for many neurological affections. Increasing evidence supports the implication of this molecule in prevalent neurodegenerative diseases, such as Parkinson’s disease, Alzheimer’s disease, or amyotrophic lateral sclerosis. The mechanisms proposed for its neurotoxicity mainly center around the increased quantities of nitric oxide that are produced in the brain, their cause, and, most importantly, the pathological metabolic cascades created. These cascades lead to the formation of neuronal toxic substances that impair the neurons’ function and structure on multiple levels. The purpose of this review is to present the main causes of increased pathological production, as well as the most important pathophysiological mechanisms triggered by nitric oxide, mechanisms that could help explain a part of the complex picture of neurodegenerative diseases and help develop targeted therapies.

## 1. Introduction

The nitric oxide/nitric oxide synthase (NO/NOS) system is one of the most studied biological systems involved in the pathogenesis of various diseases, including several neurodegenerative diseases. NO is an important signaling molecule that plays a key role both in the central nervous system (CNS) and in the peripheral nervous system functions, having a multitude of roles, such as in neurogenesis and neurodevelopment. Due to its abilities to influence the expression of genes, the synthesis of cellular DNA, the generation of action potentials, and of neurotransmission [1], the study of NO/NOS system involvement in neurodegenerative processes can provide essential elements to understanding the mechanisms of these diseases. The cyclic GMP (cGMP) pathway’s activation, for example, is an important mechanism for NO-mediated neuronal activity, such as synaptogenesis, neuron-glia interactions, dendritic and axonal growth, and ultimately, memory formation [2,3,4]. Another important process in which NO has a key role is S-Nitrosylation, a redox-mediated post-translational modification that regulates protein function through a covalent reaction of NO-related species with a thiol group in several protein families, which is essential for their proper function [5,6].

The contribution of NO/NOS in the aging phenomenon, often associated with neurodegenerative processes, is also important. The aging phenomenon is associated with multiple enzyme dysfunctions, in addition to other vascular or cerebral tissue pathological processes, significantly disrupting the functionality of the NO/NOS system. An important mechanism of senescence is linked to NO’s capacity to perform post-translational modifications of protein functions by S-Nitrosylation. Through the peroxynitrile formed during the aging process, NO exert a harmful effect on all components of cellular membranes, leading to cellular death. In the CNS, this process contributes to neurodegeneration, neuronal death, and specific dysfunctions. On the other hand, protein S-Nitrosylation is an irreversible process that can essentially contribute to pathologic accumulation of specific proteins in nervous tissue, triggering and maintaining (by associated inflammation) the neurodegenerative process in Parkinson’s disease (PD) and Alzheimer’s disease (AD) [7]. By these mechanisms, aging is a process that additionally contributes to the acceleration of neuronal death [8]. Understanding the basic mechanisms that determine the increase or decrease in NO synthesis can lead to deciphering the neuronal dysfunctions that are associated with neuronal death. The details of these mechanisms can initiate research into the effectiveness of therapies with a substantial contribution to stopping/slowing down neuronal death. The aim of this review is to create a comprehensive overview of the current information in the literature regarding the involvement of the NO/NOS system in the pathogenesis and progression of neurodegenerative diseases such as PD, AD, and amyotrophic lateral sclerosis (ALS). Additionally, since multiple paths converge and often interconnect, and as such cannot necessarily be viewed in isolation, we aim to create a comprehensive understanding of the interplay between those paths and the cumulative effect they create.

The scientific value of this work stands from the parallel investigation of the three diseases (PD, AD, and ALS, respectively), which allows the comparison of the common pathophysiological mechanisms but also shows where those paths diverge, thus enabling the exploration of future unique therapeutic concepts by comprehensively covering most, if not all, mechanisms involved. 

### 1.1. NO Formation, NOS Function, and Reactive Nitrogen Species Formation

Some key enzymes, which belong to the NOS family, contribute to NO synthesis. Their function is involved in both deficient or excessive synthesis of NO, which is an important factor in the pathogenesis of some neurodegenerative diseases of the central nervous system. The different NOS isoforms, including endothelial NOS (eNOS), neuronal NOS (nNOS), and inducible NOS (iNOS), can influence neurodegenerative pathology differently depending on the level of molecular dysfunctions or affected cellular site. The activity of nNOS and eNOS, both of which are present in neuronal tissue all throughout the brain, is highly dependent on intracellular Ca^2+^ levels, especially nNOS, which is linked to N-methyl-d-aspartate receptors (NMDAR). iNOS is not normally expressed in the brain, but can be induced in the microglia by lipopolysaccharides, cytokines, or any other form of inflammation. This particular enzyme produces high levels of NO when activated and for long periods of time [9,10,11,12]. The substantia nigra contains a large population of microglia, thus preferentially affecting the particularly vulnerable dopaminergic neurons in that region [13]. Mitochondrial NOS (mtNOS) is found in the mitochondrial inner membrane and could be of key importance in oxygen uptake and apoptosis through cytochrome c release [14]. The direct toxic effect of NO radicals has been overestimated over the years in the scientific literature as a result of researchers utilizing milimolar solutions of NO and extrapolating those reactivity results for the reactivity of NO in vivo [15]. However, NO reactivity scales with the square of its concentration for the formation of nitric dioxide (NO_2_), a powerful oxidizing agent. Thus, for in vivo concentrations of 0.1 μM to 10 μM, the direct conversion of NO to NO_2_ is irrelevant, and other reactions, such as potent reactive nitrogen species (RNS) generation and S-Nitrosylation, are the basis of NO toxicity [15].

**Figure 1:**
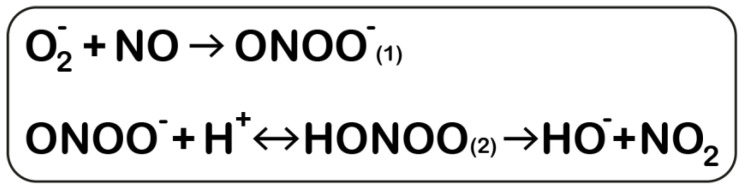
Degradation reaction chain of peroxynitrile (1) through the peroxynitrous acid (2) intermediate.

Peroxynitrile molecules, which are the result of NO reacting with superoxide radicals, can undergo protonation to form an unstable acid that quickly decays to form two reactive species with the reactivity of hydroxyl radicals and NO_2_ radicals, respectively [15] (Figure 1). Direct oxidation performed by peroxynitrile, with evidence for a far greater toxicity than that of other RNS, is also of concern in conditions with increased NO synthesis and reduced superoxide scavenging [15,16]. Additionally, copper-zinc superoxide dismutase 1 (SOD1) can react with peroxynitrile to form a powerful nitrating agent, which is able to nitrate aromatic amino acids. This is especially evident in the case of tyrosine, with the formation of nitrotyrosine, which can no longer be phosphorylated by tyrosine kinases [15,17].

S-Nitrosylation serves as an important modulatory activity of NO in both physiological processes, as well as pathological mechanisms. In the event of excessive RNS production, S-Nitrosylation plays a key role in the creation of aberrant S-nitrosylated proteins (SNO), and the misfolding and aggregation of such SNOs [5,6]. Thus, S-Nitrosylation can lead to mitochondrial impairment, synaptic damage, and, in the end, cellular death through apoptosis [6,9,18].

### 1.2. NO/NOS in Cellular Life

The identification of NO as a key endothelium-derived-relaxing-factor (EDRF) and the discovery of the synthesis of NO from L-arginine opened new ways of deciphering physiological pathways of utmost importance. Among these are the maintenance of vascular tone, the mediation of cellular defense, and at the level of the central and peripheral nervous system, the mediation of neurotransmission. The production of reactive oxygen species (ROS) at the mitochondrial level is of particular importance for respiration and cell life, thereby mediating cell survival and death [19]. Due to the energy-intensive activity of neuronal synapses, energy-producing mitochondria are essential for the integrity of synapses and appropriate neural synaptic function [6]. The synthesis of ATP via the mitochondrial respiratory chain comes at the cost of producing superoxide anions [20,21]. Intramitochondrial antioxidant systems scavenge this radical to avoid oxidative damage or generation of other reactive species such as RNS, which can lead to impaired ATP production [22,23].

Brain homogenates have been shown to contain L-arginine, which can be utilized in the production of NO and L-citrulline. Through the activation of NMDAR by glutamate [7,24,25], the formation of NO from L-arginine is amplified, due to the elevation of intracellular Ca^2+^ levels and subsequent activation of Ca^2+^ dependent nNOS and eNOS [26]. NO acts as an inhibitor of the noradrenergic, noncholinergic pathway (NANC) in the peripheral nervous system, named nitrergic neurotransmission [27]. The nitrergic pathway acts together with the noradrenergic and cholinergic pathways, contributing to the propagation of the action potential in the peripheral nervous system. The noradrenergic and cholinergic pathways are controlled and counteracted by NO [27].

Adequate NO synthesis is highly important for proper memory formation [28]. As a gas with high diffusion capacity, NO is able to diffuse from the postsynaptic ending to the presynaptic one, and independently trigger the release of neurotransmitter vesicles. As a result, an activation loop will form, termed long-term potentiation (LTP), which is the physiological mechanism of learning and memory [2,7,24]. The omnipresence of NMDAR in the central nervous system cements their importance, and by extension [29], NO’s importance in synaptic formation and modulation, memory, and learning. In recent years, great interest was shown towards these receptors, given their implication in certain neurodegenerative diseases, such as ALS, AD, and PD, which will be further discussed in their respective chapters [30]. The half-life of NO depends on its concentration [31], and the production can be modulated and inhibited by the administration of N-monomethyl-L-arginine (L-NMMA), which can act at the level of the nervous system as well [32].

**Figure 2:**
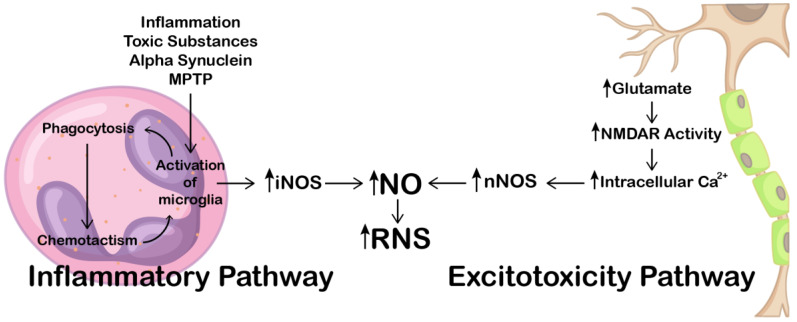
Mechanisms implicated in elevated NO generation. On the right, the excitotoxicity pathway begins from excess glutamate, which through coupling with NOS enzymes via Ca^2+^ leads to an increase in NO production. On the left, the inflammatory pathway begins with any form of acute or chronic inflammation, which through the activation of inflammatory cells, including microglia, leads to the formation of de novo iNOS. In a Ca^2+^ independent manner, iNOS increases the production of NO. Both pathways culminate in the common end result of increased RNS formation. Abbreviations: iNOS, inducible nitric oxide synthase; MPTP, 1-methyl-4-phenyl-1,2,3,6-tetrahydropyridine; NMDAR, N-methyl-d-aspartate receptors; nNOS, neuronal nitric oxide synthase; NO, nitric oxide; RNS, reactive nitrogen species; ↑ increase; ↓ decrease. References: [9,10,11,12,15,33,34].

The main pathways involved in elevated NO generation have been summarized in Figure 2.

### 1.3. NO/NOS Influence on the Blood Brain Barrier Permeability

The blood-brain barrier (BBB) is a structure with highly selective permeability only for certain molecules, thus protecting the brain from the toxic effects of multiple substances in the blood that result from normal metabolic processes or from inadequate clearance associated with various diseases. The protecting role of the BBB is ensured by the tight junctions between the vascular endothelial cells, a structure that can become more permeable by the action of NO as a result of its vasodilatation effects [35]. In certain situation, this effect can excessively increase the BBB permeability, allowing different molecules to permeate and subsequently undergo metabolic transformations in the brain tissue, with the formation of pathological deposits that can initiate degenerative processes and neuronal death. Simultaneously, ionic, osmotic and vasogenic cerebral edema can occur, which can also have a detrimental effect on the structure and function of the CNS [36]. nNOS may play a major role in BBB disruption [37]. For this reason, nNOS inhibitors may have beneficial effects in cerebral ischemia or in neurodegenerative processes by reducing BBB permeability. It has been suggested that the burst of both eNOS and nNOS, following acute cerebral ischemia, mediates the increase in NO production. The consequence is the occurrence of vasogenic cerebral edema with brain tissue damage [36]. The production of NO occurs in two waves, the first of which is characterized by the transient increase in NO synthesis, and the second one by an increased synthesis of NO that lasts up to 4–7 days. This increase in NO synthesis is correlated with elevated intracellular Ca^2+^ levels in both waves [38,39]. The local inflammatory response, through the production of pro-inflammatory cytokines that stimulate iNOS, continues to generate elevated levels of NO, even if reperfusion of the ischemic tissue occurs. Thus, an inhibitor such as Nitro-L-arginine methyl ester (L-NAME) can be employed to stop both vasodilation and BBB permeability loss [37]. On the other hand, NO and NOS inhibitors can constitute triggers for the development of focal cerebral ischemia, which with sufficient time duration can lead to neuronal death with permanent lesions and neurological dysfunctions [1,40]. Moreover, during reperfusion, excessive NO production occurs because blood flow is re-established, and with it, the supply of oxygen, and toxic radicals of oxygen and nitrogen, renewed [41]. The result is an increase in cerebral edema. That is why early inhibition of NO formation following cerebral ischemia can be a way to prevent the loss or reduce the permeability of the BBB and prevent the permeation of harmful substances in the brain tissue, which can become subsequent triggers for neurodegenerative processes. It is already known that cerebral ischemic changes that occur simultaneously with the senescence process can lead to the initiation or acceleration of pathological processes that are the basis of neurodegenerative diseases such as PD and AD [42,43].

## 2. Parkinson’s Disease

PD is a neurodegenerative disorder caused by the specific death of dopaminergic neurons in the substantia nigra pars compacta, which manifests clinically by tremor, brady- or akinesia, rigidity, gait impairment, as well as other motor and non-motor symptoms [6,44,45]. As life expectancy increased, it has become the most frequent motor disorder and, after AD, the second most frequent neurodegenerative disease [6,9,44]. PD pathophysiology is not completely elucidated, but the disease can either be of genetic cause, which accounts for 5–15% of cases, or can have a multi-factorial basis (non-hereditary form) [44]. Many exogenous and endogenous factors can initiate and influence the onset and course of PD. For example, genetic factors, oxidative stress and impaired autophagy, together with inflammation (which is ubiquitously associated with lesions of the central nervous system) can converge and influence cellular death [46]. The genetic form of PD has an autosomal recessive or dominant transmission, an early onset, and is characterized by mutations in or deletions of certain genes such as Parkin, Dj-1, protein disulfide isomerase (PDI), PTEN-induced kinase 1 (PINK1), or SNCA, which have been shown to be involved in protein degradation, antioxidant mechanisms, cellular defense, maintaining mitochondrial integrity and function [6,9,14,44]. Therefore, alteration of the specific proteins encoded by these genes, especially through post-translational modifications, could be involved in non-hereditary PD pathogenesis [9]. An important risk factor for PD development, which increases the risk by two to three times compared to controls, is first-degree relatives suffering of PD [44,47]. The non-hereditary PD form, which has a higher frequency, can arise through complex interactions between genetic predispositions and a multitude of environmental factors, such as toxic substances (pesticides), head trauma, as well as through the normal process of neuronal ageing [6,14,44,47,48]. The most important risk factors for non-hereditary PD are age and sex, males being more affected. Interestingly, protective factors have also been suggested, such as coffee, cigarette smoking, statins, and Ca^2+^-channel blockers [44,47]. The implication of the NO/NOS system in PD pathogenesis could be of particular interest for therapy perspectives.

### 2.1. Implications of NO and NOS in Parkinson’s Disease

NO can have damaging effects through a multitude of mechanisms, depending on its concentration and the redox state of the cell [6,14]. The final effects of this imbalance are apoptosis, necrosis, or autophagy of the affected neuronal population, which are important mechanisms in PD pathogenesis [18]. Experimental studies have shown a clear implication of NOS in PD pathophysiology, when administrating toxic PD-inducing substances and NOS inhibitors [18,49]. For example, several studies have focused on 1-Methyl-4-Phenyl-1,2,3,6-Tetrahydropyridine (MPTP) [14,50,51,52] or 6-Hydroxydopamine (6OHDA), both specifically affecting the dopaminergic neurons, leading to the development of a PD-like syndrome [14,53]. The results showed that MPTP induced upregulation of iNOS, hinting at the idea that MPTP activates microglia. Moreover, administration of substances that decrease or inhibit NOS function protected against MPTP-induced neurotoxicity [54,55]. In another study, mice with knockout nNOS and iNOS genes were also protected from MPTP [51,55,56,57]. As for 6OHDA, prior administration of NOS inhibitors offered neuronal protection [14,58,59].

Further evidence for NOS implication in PD development came from post-mortem studies performed on the brains of PD patients. Eve et al. [60] found that mARN for NOS was increased in the subthalamic nucleus, specifically the dorsal two thirds, but decreased in the putamen. Another study found that the iNOS gene was upregulated in the substantia nigra in PD patients but was normal in the striatum compared to controls [61]. Nitrosyl complexes, which are created when an NO radical binds to a transitional metal [62], were also found to have a higher concentration in the substantia nigra of PD patients [63]. Overexpression of nNOS was noted in circulating neutrophils [64], and upregulation of the iNOS gene was noted in the microglial cells of PD patients [65]. Certain mutations in NOS genes have also been linked to an increased predisposition towards familial PD development [14,66]. The results of these studies clearly show the implication of NO in the pathogenesis of this disease.

The mechanisms by which NOSs are activated and/or upregulated are not completely clear. Several factors can be incriminated: inflammation can trigger microglia activation, thus increasing iNOS activity [9]. Glutamate increase in the form of excitotoxicity activates both synaptic and extrasynaptic NMDARs [33], triggering intracellular Ca^2+^ levels to rise, activating nNOS [10]. mt NOS can be stimulated by dopamine, also explaining why dopaminergic neurons are preferentially affected in PD [67]. Lastly, NO stimulates its own production by Ca^2+^ release into the cytoplasm from the endoplasmic reticulum, the mitochondria, or through activation of the NMDAR [18]. Activated NMDARs also lead to increased ROS formation [6].

### 2.2. Importance of S-Nitrosylation in Parkinson’s Disease

S-Nitrosylation is believed to be of particular importance in PD pathogenesis. Certain key proteins, whose functions were linked mainly to mitochondrial function, such as PINK1 and Parkin, as well as cytoprotective function, such as DJ-1, may have an important role in PD pathogenesis. Mutations of their genes have been linked to early onset PD [6,9,14], autosomal recessive transmission, implying that loss of function in these proteins leads to neurodegeneration [68]. Thus, aberrant SNOs formed by S-Nitrosylation of those key proteins might have a similar effect.

DJ-1 is a small (189 amino acids) protein found throughout the cell and has mainly cytoprotective roles, promoting cell survival and limiting cell death. S-Nitrosylation could be the culprit for this protein’s alleged loss of function in PD, given that DJ-1 has three cysteine residues (c46, c53, c106) that could be nitrosylated. C106 is the most conserved site of the three because substitutions of this amino acid lead to loss of function. Therefore, the S-Nitrosylation of this particular site could lead to protein malfunction [9].

Parkin is a ubiquitin involved in protein degradation, mitochondrial function, and endoplasmic reticulum (ER) stress. Through proteolysis via the proteasome, degraded and no longer necessary proteins are disassembled, limiting protein accumulation in the cytosol. Parkin also regulates mitochondrial function through mitophagy, thus limiting ROS formation [9,69]. Parkin modulates ER stress, which, although can also be activated by other mechanisms, happens if misfolded proteins accumulate in the ER lumen. The ubiquitin function of Parkin prevents this accumulation [70]. Parkin has also been shown to inhibit p53 expression, thus preventing apoptosis in the dopaminergic neurons. Several studies suggested a possible implication of damaged Parkin in PD pathophysiology. Chung et al. demonstrated increased quantities of S-nitrosylated Parkin both in vivo and in vitro, following exposure to certain PD-inducing substances (such as MPTP) and sampling human brain tissue of PD patients. The same study showed a decreased capacity of S-nitrosylated Parkin in promoting cell survival [71]. Loss of Parkin function was hinted by Sunico et al, whose results showed a decreased activity of S-nitrosylated Parkin in inhibiting p53. [72]. Yet another experimental study showed protein accumulation and mitochondrial dysfunction following Parkin S-Nitrosylation [73]. Therefore, it is possible that if this protein becomes non-functional due to S-Nitrosylation, protein aggregates would form, ROS would increase, ER stress would develop, and finally, p53 would not be inhibited anymore, all these mechanisms eventually leading to apoptosis [9,70]. These hypotheses need further studies to show the exact extent of which malfunctioning Parkin leading to PD.

PINK1 is a serine/threonine kinase involved in mitochondrial homeostasis and mitophagy, as well as inhibition of ROS-induced apoptosis [74]. It works by activating Parkin, and together they mediate mitochondrial fusion and fission. Sircar et al [9] demonstrated (using an in-vitro model) that administration of a NOS inhibitor alongside vancomycin (which accelerates the formation of S-nitrosylated PINK1) reduced the negative impact on the cell, implying the role of NOS in PINK1 S-Nitrosylation. Loss of function in PINK1 due to S-Nitrosylation (C568 was identified as the most likely site) has been shown to lead to neuronal death in vitro, thus establishing a basis for PD pathogenesis [9].

PDI is a cellular defense protein which boosts chaperone and isomerase activity, increasing its activity in case of ER stress [70]. However, S-Nitrosylation can block this neuroprotective effect because it impairs PDI’s capacity to address protein misfolding. Significant quantities of SNO-PDI are seen in brains of people who have sporadic PD. These findings imply that PDI is abnormally S-nitrosylated during neurodegeneration, and this post-translational modification may speed up the disease’s progression [6].

### 2.3. Peroxynitrile’s Role in Parkinson’s Disease

Given the fact that neurons, in general, and dopaminergic neurons, in particular, are especially susceptible to oxidative stress, imbalances in ROS and RNS formation can lead to cell death [6]. Through the reaction of NO and superoxide previously explained, peroxynitrile is formed, a reaction accelerated due to the increased creation of superoxide during dopamine metabolism [7,70]. Peroxynitrile is thought to be one of the most damaging molecules in PD pathogenesis due to its high affinity for mitochondrial complexes (I, III, IV), mitochondrial DNA, and nuclear DNA, leading to mitochondrial and DNA-mediated apoptosis but also to necrosis and autophagy [9,67]. Peroxynitrile was also proven to reduce the uptake of dopamine in dopaminergic neuron by inhibiting the presynaptic dopamine transporter [7]. NO also mediates the release of iron from transferrin, leading to ROS formation and activating ferroptosis, a particular type of apoptosis modulated by high iron concentration [13,75]. Increased NO and peroxynitrile can damage DNA, either directly or by inhibiting its synthase and repair, resulting in cell apoptosis [11,14].

### 2.4. Mitochondrial Damage in Parkinson’s Disease

By far, the most damaging effect of high levels of NO is mitochondrial impairment, considered to be one of the main causes of neuronal death in PD [9,67,70]. Mitochondria are extremely important cell organelles, especially to neurons, given their high demand of ATP and their sensibility to its decrease. Therefore, their impairment could have catastrophic consequences for the neurons [67]. Inhibition of the respiratory chain leads to the formation of ROS, mitochondrial permeability, cytosol Ca^2+^ imbalance, and cytochrome c release, leading to metabolic cascades and positive feedback loops, which ultimately end with apoptosis of dopaminergic neurons [14,67,70]. The increase in cytosolic Ca^2+^ also triggers ER stress, leading to the accumulation of degraded proteins [70].

### 2.5. Dopamine Metabolism and NO/NOS System in PD

Given that dopaminergic neurons are the main cells affected in PD, it is important to discuss certain physiological characteristics and their connections with NO/NOS system. These neurons, being part of the substantia nigra, are highly important in extrapyramidal pathways, controlling and modulating movement [76]. Loss of their function will lead to persistent alterations in the nigrostriatal pathway, ultimately generating the characteristic symptoms observed in PD [18]. Although it is unclear why these neurons are specifically targeted in PD, some hypotheses have been proposed. Inflammation and excessive ROS /RNS formation, as well as decreased levels of antioxidants, have been linked to cell death [48,77]. Dopaminergic neurons are particularly sensible to oxidative stress, making them more vulnerable to inflammation and high concentrations of ROS [6]. Excessive ROS can also be produced via mitochondrial dysfunction, which has been proposed in PD pathophysiology. Recent studies have linked mutations in DJ-1, Parkin, and PINK1, genes involved in mitochondrial function, to early onset PD [45]. Dopaminergic neurons contain both synaptic and extrasynaptic NMDAR, making them highly susceptible to glutamate excitotoxicity [33]. High levels of glutamate will bind to an increased number of NMDARs, inducing a rise in Na^+^ and Ca^2+^ intracellular levels. While Na^+^ leads to neuron swelling, which is reversible, Ca^2+^ mediates irreversible cellular injuries, initiating the process of apoptosis [33]. Ca^2+^ -induced apoptosis is triggered by imbalances in this ions’ homeostasis, mainly through increased intracellular quantities. By binding to calmodulin, its main cofactor, Ca^2+^ activates certain protein kinases and phosphatases, which regulate gene transcription, thus upregulating apoptosis [78].

It is worth mentioning that high intracellular Ca^2+^ levels will lead to NO formation [10,79], and that NO can activate NMDAR by inhibiting dopamine reuptake [18], thus creating a positive feedback loop. NMDARs are also involved in secondary excitotoxicity, even in the absence of glutamate. A decreased ATP level (due to NO-induced mitochondrial impairment) can inhibit the Na^+^/K^+^ pump, leading to partial cell depolarization and triggering the consistent activation of the NMDARs [67]. 

Characteristically, in most cases of PD, misfolded alpha-synuclein (aSYN) protein inclusions called Lewy bodies (LB) are present in the cytoplasm [44]. These aggregates may also be responsible for neuronal cell death, given their presumed toxicity [80]. As mentioned above, their main constituent is misfolded aSYN proteins, which are coded by a mutant the SNCA gene. Mutations and duplications of this gene were linked to hereditary PD, thus showing a connection between aSYN and PD [45]. 

Dopamine, or its metabolites, can also be incriminated in PD pathogenesis, playing an important role and explaining why the dopaminergic neurons are specifically affected in this disease. It must be noted firstly that tyrosine hydroxylase, an enzyme involved in dopamine formation, and NOS are both located inside the dopaminergic neurons [14]. Several mechanisms in which excessive NO levels lead to the abnormal dopamine metabolism and vice versa have been summarized in Table 1 below:

Dopamine oxidation leads to dopamine-quinone, which has toxic effects on mitochondrial metabolism, causing a decrease in ATP and glutathione and an increase in ROS [14,82]. Another byproduct of dopamine metabolism, 3,4-dihydroxyphenylacetic acid (DOPAC), is formed in the mitochondrial matrix. Oxidation of DOPAC by NO (formed by mtNOS) inhibits cytochrome c oxidase (complex IV), damaging the mitochondria [14,67]. DOPAC and NO acting synergistically were shown to decrease glutathione, resulting in a higher ROS concentration [67]. Finally, dopamine metabolite superoxide anion [82] combines with NO to form peroxynitrile, which leads to DNA damage, protein misfolding, mitochondrial inhibition, and finally, cell death [14,67,70].

**Figure 3:**
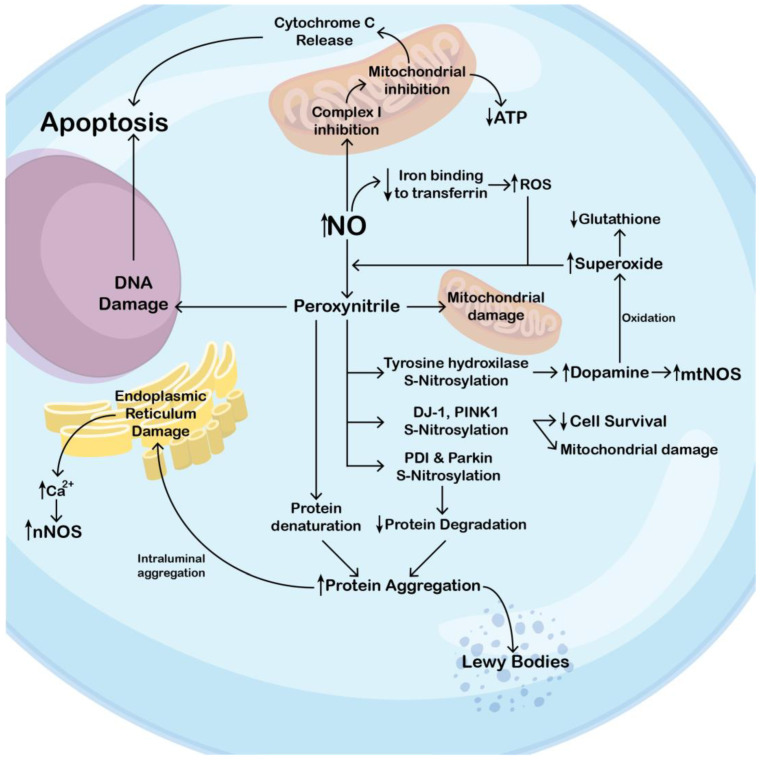
High concentrations of NO can negatively impact cellular function either directly or through peroxynitrile. Direct actions of NO include mitochondrial inhibition through complex I inhibition and release of bonded iron from transferrin. Mitochondrial inhibition through complex I inhibition results in lowered ATP production, which increases mitochondrial permeability, leading to cytochrome c release, thus activating apoptosis. Through the release of bonded iron from transferrin, NO increases the reactivity of iron ions, and subsequently, the formation of ROS. In combination with superoxide, NO forms peroxynitrile, a toxic compound with many deleterious effects. Peroxynitrile irreversibly damages mitochondria, damages DNA and induces apoptosis, and S-Nitrosylate key cellular proteins. S-Nitrosylation of tyrosine hydroxylase leads to an increase in its function and therefore increased dopamine synthesis. As a result of increased dopamine levels, mtNOS activation will lead to the formation of extra NO, and the excess of dopamine metabolism will increase superoxide concentrations and decrease glutathione levels, leading to the formation of more peroxynitrile. S-Nitrosylation of other proteins, such as DJ-1 and PINK1, whose main roles revolve around mitochondrial integrity and cell survival, could impair these exact roles, damaging mitochondria and promoting cellular death. Another two proteins, PDI and Parkin, which are involved in protein degradation, could lose their function due to S-Nitrosylation, increasing intracellular protein accumulation, and formation of Lewy bodies. Protein aggregation inside the endoplasmic reticulum will lead to ER stress, impaired function, and release of Ca^2+^, activating nNOS, increasing NO levels. Abbreviations: ATP, adenosine triphosphate; DNA, deoxyribonucleic acid; ER, endoplasmic reticulum; mtNOS, mitochondrial nitric oxide synthase; nNOS, neuronal nitric oxide synthase; NO, nitric oxide; PDI, protein disulfide isomerase; PINK1, PTEN-induced kinase 1; ROS, reactive oxygen species; ↑ increase; ↓ decrease. References: [7,9,11,13,14,15,44,67,69,70,74,75,82,83].

The schematic bellow (Figure 3) represents an overview of the previously described pathways in which NO is involved, as well as the interplay between them.

## 3. Alzheimer’s Disease

AD is a neurodegenerative disorder associated with progressive cognitive deficits due to the accumulation of intracellular neurofibrillary τ tangles and amyloid β (Aβ) plaques [2,22,84,85,86,87,88]. Amyloid precursor protein (APP) uptake from the plasma membrane to the endocytic compartment is further degraded by β and γ secretases, resulting in Aβ [22,84,88,89]. An elevated level of Aβ plays an important role in the pathogenesis of AD [22,84]. There are two types of AD: familiar or early-onset AD (fAD) and sporadic or late-onset AD (sAD) [88,90]. The precise cause of sAD is currently unknown; however, advanced age and inheritance of the apolipoprotein ε (Apoε) gene’s 4 alleles can both be important risk factors [88]. Numerous genetic mutations have been discovered in fAD [88,90]. Presenilin-1 or Presenilin-2 gene mutations (PSEN1, PSEN2) are the most frequent fAD mutations, and APP-encoded amyloid precursor protein duplications and mutations are also associated with the disease [88,90]. 

NO has been discovered to have an important role in the pathophysiology of AD. Excessively high levels of NO, whether caused by nNOS activation by excito-toxicity, induction of iNOS in glial cells, or eNOS activation in vascular tissues, may be harmful, especially if it occurs simultaneously with an increase in the production of free radicals [3,84,91]. Thus, NO has been shown to have three primary physiological effects that have all been linked to AD: a. direct S-Nitrosylation of protein cysteine residues, b. protein tyrosine nitration, and c. signaling via the soluble guanylate cyclase and the cyclic guanosine monophosphate (cGMP) pathway, which will be further discussed [2,6,7].

### 3.1. S-Nitrosylation in Alzheimer’s Disease

In sAD, a variety of proteins essential for synaptic survival and function are abnormally S-nitrosylated, causing synapse loss and neurodegeneration [6]. The Apoε gene encodes a group of lipoproteins essential for maintaining CNS cholesterol homeostasis [6,91,92]. There is evidence that Apoε4 is a significant genetic risk factor for sAD developing [6,93]. Moreover, in the human hippocampus, both Apoε2 and Apoε3 can be S-nitrosylated [6,91], as shown in an experimental cell culture study [94], which may result in altered protein conformation, decreasing its affinity for low-density lipoprotein (LDL) receptors [6]. Samples from both cell culture and human hippocampus revealed that Apoε2 and ε3 can be S-nitrosylated, suggesting a potential role of this post-translational modification in sAD [6]. Moreover, there is an isoform-specific difference for Apoε in microglial NO production: mice expressing the Apoε4 protein isoform have a greater NO production than mice expressing the Apoε3 protein isoform, and both neurons and macrophages from Apoε4 transgenic mice exhibit a similar increase in the uptake of arginine [91]. In AD patients with the ε4 allele, increased cerebral oxidative stress has been seen [91].

### 3.2. Protein Tyrosine Nitration

Within areas of neurodegeneration in AD, nitrotyrosine levels are increased in as-trocytes, blood vessels, and cytoplasm of cerebral cortex neurons [91]. The extensive prevalence of nitrotyrosine immunoreactivity shows that chronic oxidative damage causes generalized oxidative stress that contributes to the pathogenesis of AD [91]. It is interesting to note that post-mortem examination of brain tissue from AD patients revealed elevated levels of 3-nitrotyrosine staining, a molecular marker of peroxynitrile production in individuals with the ε4 allele [23,95].

### 3.3. Signaling via sGC and cGMP Pathway

cGMP is one of the most common and widely spread secondary messengers throughout the mature brain. Being mainly synthesized by soluble guanylyl cyclase (sGC) and degraded by a wide range of phosphodiesterases (PDEs), its concentration is a complex balancing act that changes with age [4]. 

It has been found that cGMP levels decline with age [4,96], and yet, this decline could be reverted in a rat model by the use of 3-isobutyl-1-methylxanthine (IBMX), a non-selective PDE inhibitor [97]. This suggests that the cGMP decline is not a consequence of impaired synthesis but rather a consequence of increased degradation.

This decline in cGMP concentrations can lead to impaired NO anti-apoptosis responses. Through cGMP, NO can promote the phosphorylation and inactivation of pro-apoptotic proteins, inhibiting the process [98]. Lower cGMP concentration due to increased degradation would lead to a decreased anti-apoptotic effect, an increase in NO concentration, and in the end, reactions with superoxide and formation of peroxy-nitrile [99].

### 3.4. NO in Memory and Learning

In a healthy brain, NOS enzymes play an important role in memory and learning development [84,100]. 

LTP is a fundamental process for memory formation in the hippocampus, the center responsible for memory and learning [4,7,101]. Being a form of synaptic plasticity, it has been shown that LTP is influenced by NMDAR through repetitive stimulation [102]. This repetitive stimulation, which in other cases has been shown to be detrimental due to excitotoxicity, is beneficial in the case of LTP, strengthening its connections [103]. 

Previous studies have linked the well-functioning of LTP to the NO/cGMP pathway [4]. It has been shown through multiple experiments [104,105] that NOS inhibitors lead to impaired learning and memory through LTP impairment, thus implying a positive role of NO in these processes [101].

However, due to the decreased number of NMDAR, as a result of both aging [96,97] and AD pathophysiology [106], it was postulated that NO synthesis is upregulated, in an attempt to maintain the LTP functioning, but ultimately leading to neurotoxicity [107,108].

In AD, the hippocampus is one of the main affected sites [7]. Although the hippocampus has high levels of amyloid precursor protein (APP) expression, direct interactions between the Aβ and neuronal membranes and receptors, such as NMDAR, cause Aβ production, abnormal Ca2+ influx, and nitrosative stress [3,7,101]. This increases τ phosphorylation, which makes the hippocampus more susceptible to nitrosative damage [3,7,90,101].

### 3.5. nNOS, eNOS and Cerebral Blood Flow in Alzheimer’s Disease

Due to the extensively high energy demands coupled with the paradoxically low energy storage, the brain requires continuous and dynamically regulated blood supply in order to maintain proper function [109]. A precise and prompt blood flow control that can dynamically increase the supply to areas of intense neuronal activity is thus required. This process, named neurovascular coupling (NVC), is accomplished by the communication between active neurons and the cells that form the neurovascular compartment [110] through a multitude of molecular pathways. Among those pathways, NO plays a ubiquitous key role in the process and is essential for the development of the neurovascular response. 

Through the formation of a supramolecular complex with NMDAR, nNOS activity becomes regulated by glutamate, through Ca^2+^ intracellular influx [25]. As a result of NMDAR activation. Properly dimerized nNOS will proceed to produce NO, however, under the condition of disrupted dimerization, the enzyme catalyzes the uncoupled oxidation of nicotinamide adenine dinucleotide phosphate (NADPH), with the production of superoxide instead [111]. One of the noteworthy factors that can lead to reduced nNOS dimerization is tetrahydrobiopterin (BH4) availability, which has been noted to be reduced in AD patients [112]. 

Physiologically, NO produced in neurons with intense activity can diffuse intercellularly, reaching the smooth muscle cells in the tunica media of adjacent cerebral blood vessels. There, through the activation of sGC and the formation of cGMP, NO exerts a vasodilatory effect through the relaxation of smooth muscles cells [113]. While the increased cGMP degradation described in the previous chapter can decrease NO’s effectiveness, a more substantial decrease can be attributed to NO scavenging produced by superoxide, and subsequent formation of peroxynitrile [25]. Thus, nNOS dysfunction, in the form of disrupted dimerization, can decrease the vasodilatory effectiveness of NO by increasing superoxide’s NO scavenging, while also increasing NO’s toxic effects through the increased production of peroxynitrile.

Furthermore, Aβ can reduce the responsiveness to vasodilatory molecules such as NO, resulting in decreased cerebral blood flow (CBF) [114], which in turn will promote the formation of more Aβ, creating a vicious cycle that will sustains the progression of the disease. However, Aβ accumulation and involvement in the progression of the disease occurs in the later stages of the disease, with NVC changes preceding both NO dysregulations and Aβ accumulation [25,115]. 

In summary, nNOS dysfunction prompts a vicious cycle in which NO’s vasodilatory effectiveness is impaired, while its toxic effects, through the increase in peroxynitrile, are amplified. Furthermore, peroxynitrile can further sustain the progression of the cycle by oxidizing BH4, inhibiting sCG expression and activity, and promoting structural alteration in cerebral blood vessels [25,116].

eNOS is the constitutive NOS form found in endothelial cells, requiring similar cofactors and conditions as nNOS to function properly. Unlike nNOS, eNOS is activated as a result of Ca^2+^ influx caused by shear forces experienced by the endothelial cells [91,117]. After activation of eNOS, the NO produced is able to diffuse to the underlying smooth muscle cells and trigger relaxation and vasodilation [91,117]. 

While experimental data show that eNOS expression is upregulated in AD models [118,119], its activity can be decreased by uncoupling caused by peroxynitrile or a BH4 deficit [91,117]. The effectiveness of NO can be reduced by the production of superoxide by the uncoupled enzyme [25,117]. Additionally, Aβ accumulation can reduce the responsiveness of vessels to NO produced by eNOS, similar to the case of nNOS. Of importance might be the fact that increased eNOS expression is seen in the same locations where elevate nitrosative stress is quantified by increased nitrotyrosine levels [91], which could be caused by an increase in expression as a response to reduced activity due to uncoupling. However, additional research is needed.

### 3.6. eNOS and Inflammation in Alzheimer’s Disease

To cause vasodilation and subsequent leukocyte influx, eNOS produces NO [7]. However, under physiological circumstances, this reduces leukocyte diapedesis, which serves as an immune brake [7,22]. Platelet and leukocyte adhesion to the endothelium is suppressed by NO by promoting vascular cell adhesion molecule-1 (VCAM-1) and intracellular adhesion molecule-1 (ICAM-1), and this prevents pro-inflammatory cells and other proteins from persistently homing to the inflamed tissue [22]. This would therefore aid in resolving proinflammatory reactions [22,120]. Therefore, eNOS deficiency could lead to an increase in inflammatory agents [22,120]. The activation of microglia in the brain causes reduced Aβ clearance to further trigger inflammatory responses [22,84]. Endothelial cells forming the BBB also actively participate in the clearance of Aβ into the circulating blood, and dysfunction of BBB clearance is considered an important mechanism in the pathogenesis of AD [84,120]. The receptor for advanced glycation end products (RAGE) mediates the influx and re-entry of circulating Aβ across the BBB into the brain [84,89,90,120]. On the other hand, the low-density lipoprotein receptor-related protein-1 (LRP) mediates Aβ clearance from the brain to circulating blood [84,90,120]. The liver and kidneys are responsible for the systemic clearance of free Aβ and of complexes between circulating soluble LRP and Aβ [84,90,120]. 

Existing evidence indicates that there is a significant negative correlation between the expression of eNOS in brain capillaries and AD lesion burden [90]. These findings imply that eNOS could be a direct modulator of the endothelial Aβ transport proteins, including LRP and RAGE [120]. Moreover, the observed reduction in capillary eNOS expression might represent a secondary phenomenon resulting from vascular injury and loss of endothelial homeostasis caused by the progression of AD [120]. Considering this, it would be logical to hypothesize that NO deficiency triggers an inflammatory response that harms neurons and advances AD pathogenesis [22,120].

### 3.7. iNOS and Inflammation in Alzheimer’s Disease

The primary cells involved in neuronal inflammation are neurons, endothelial cells, non-neuronal astrocytes, blood-derived macrophages, and microglia, and each cell type’s dynamics and biological actions are altered in AD [84,89]. They are responsible for detecting and eliminating cellular pieces after an injury, foreign infections and protein clumps such as Aβ [4,22,89]. These immune cells and non-neuronal cells use damage-associated receptors (DAMPs) and pathogen recognition receptors (PRRs) to detect bacteria and substances that are non-self [22,89]. NO is one of the biomolecules that microglial cells produce during an injury or when reacting to foreign antigens [22]. In a chronic inflammatory response brought on by ongoing infection or even by ongoing deposition of inflammation-triggering mediators, such as Aβ in AD, there is a sustained high level of iNOS, which leads to elevated levels of NO [4,22,89]. 

Increased NO synthesis by iNOS is a key contributor to the neurodegeneration brought on by oxidative stress [4,84,89,121]. As a result, it stimulates the innate immune system and effectively stops foreign bodies from invading [84,121]. Additionally, reactive microglia and astroglial cells generate iNOS, which results in higher NO levels [84,89,121]. Due to the nitration of protein tyrosine residues, neuronal damage and death occur, therefore AD lesions are recognized as hallmarks of oxidative and nitrosative stress-induced injury [4,22,84]. Thus, the decompensation of the BBB and the harm to brain parenchymal cells are likely caused by the increased accumulation of NOS products [25,91]. There are some findings which show that removal of iNOS in transgenic AD mice or the use of iNOS inhibitors to block NO production has been shown to protect against Aβ-induced neurotoxicity, indicating that nitrosative stress may be one of the key factors mediating Aβ pathogenesis in AD [88]. In addition, some findings show that the genetic suppression of iNOS significantly protects AD-transgenic mice from premature mortality, the formation of cerebral plaques, the load of amyloids, the nitration of tyrosine proteins, astrocytosis, and microgliosis [91].

### 3.8. NO and Oxidative Stress-Associated Lipid Peroxidation in Alzheimer’s Disease

NO interacts with a variety of molecules, including lipids [4]. Unsaturated fatty acids found in membranes, such as polyunsaturated fatty acids (PUFAs), are susceptible to harmful oxidative damage from this highly reactive nitrogen species [22,91,92,122]. 

Chemically reactive aldehydes such as malondialdehyde (MDA), acrolein, 4-hydroxy-2-hexenal (HHE), and 4-hydroxy-2-nonenal (HNE) are released as a result of fatty acid peroxidation [20,22,34,122]. HNE is primarily found in membranes and interacts with membrane proteins to form adducts in neuronal cells [22,34,122]. Thus, HNE can possibly obstruct the transmission of signals along the neurons, cause neuronal damage, and result in cell death [22,34,122]. Additionally, HNE reacts with Aβ and adds covalent modifications, causing the formation of cross-linkages and ultimately aggregation, a defining feature of AD [22,34]. Acrolein has also several harmful effects, including membrane disruption, disruption of mitochondrial biogenetics, protein adduction, and DNA oxidative stress [22,34]. 

Therefore, increased NO levels brought on by inflammation may serve to trigger the emergence of additional neuronal and non-neuronal pathologies, such as cancer, in addition to worsening the disease’s progression [22].

### 3.9. NO and Mitochondria in Alzheimer’s Disease

In AD, damaged mitochondria due to Aβ are unable to maintain the energy demands of the cell, and the activity of ATP synthase is decreasing [23,122]. Thus, AD may con-tribute to mitochondrial malfunction, including excessive fragmentation, which in turn causes synaptic dysfunction, neuronal damage, and finally cell death [4,22,123]. There is growing evidence that NO disrupts mitochondrial energy dynamics by competing for oxygen with cytochrome oxidase [23]. Neuronal cellular death results from poor energy production, disrupted mitochondrial function, and a disparity in the body’s capacity to combat ROS [23,123]. Dynamin-related protein 1 (DRP1) is a GTPase involved in mitochondrial fission. S-Nitrosylation of DRP1 is increased in AD patients, thus increasing its activity through a gain of function modification [4,21]. As a result, excessive mitochondrial fission, energy depletion, and ultimately dendritic spine loss result from this SNO-DRP1 synthesis [4,6]. Moreover, peroxynitrile is suggested to cause protein aggregation through nitrosylation of membrane protein thiols in mitochondria [6,22]. This leads to the formation of pores, which would compromise the structural and organizational integrity of the mitochondrial membrane, hence resulting in the efflux of mitochondrial contents such as apoptosis-inducing factors, consequently leading to apoptotic neuronal loss [22].

## 4. Amyotrophic Lateral Sclerosis

ALS is a fatal neurodegenerative disease characterized by degeneration of both upper and lower motor neurons, resulting in dysfunction of the somatic body muscles [124,125,126]. It is one of the most common type of motor neuron diseases (MNDs) [126]. The main neuropathological features of ALS can be divided into three main classes: a. lower motor neuron affliction, described as an extensive loss of motor neurons in the anterior spinal cord and motor nuclei in the brain stem [127,128], b. upper motor neuron affliction, represented by the loss of large pyramidal cell neurons, known as Betz cells, in the primary motor cortex, and the subsequent degeneration of the lateral corticospinal tracts [129,130,131], and c. hypertrophy of glial cells, also known as reactive gliosis, in the areas of degeneration from the motor cortex and spinal cord [132,133,134].

### Involvment of NO in the Patogenisis of Amyotrophic Lateral Sclerosis

One of the most important mechanisms considered as a part of the pathogenic mechanism is represented by the imbalance between oxidative stress/antioxidant systems. While 5 to 10% of all cases of ALS are caused by genetic mutations, known as familial forms of ALS (fALS), most cases of ALS are considered to be sporadic (sALS) [125]. As a key component of antioxidant systems, SOD1 is a free-radical scavenging enzyme omnipresent in human cells. Several mutant variants of SOD1 (mSOD1) have been discovered in fALS patients, which have the tendency to misfold and form aggregates in the cytoplasm of motor neurons [135,136,137]. Additionally, misfolded wild-type SOD1 aggregates, which will be discussed later, have been incriminated as a cause for sALS as well, without any evidence of genetic mutations [138,139,140]. Aggregates composed of ubiquitinated mSOD1 have been found in fALS patients to inhibit proper protein degradation, leading to neuronal death [135,136,137], however, other mutations in protein degradation genes, such as valosin-containing protein (VCP), OPTN, TBK1, and SQSTM1, have also been found to impact the neuronal protein metabolism of ALS patients [141,142]. Cytoplasmic aggregation of wild-type SOD1 have been frequently found in sALS patients [141], due to a prion-like mechanism in which misfolded mSOD1, or more often other misfolded proteins, induce the aggregation of wild-type SOD1 in neighboring neurons [138,142]. With NO radicals being the only biological molecule known to outcompete SOD1 for the reaction with superoxide radicals [15,143], the importance of RNS species resulted from the reaction of NO with the superoxide radical becomes ever so important in conditions that affect the function of SOD1, such as mSOD1 fALS and some forms of sALS. This capability to outcompete SOD1 [143] becomes ever so important in ALS cases with mutant SOD1 or SOD1 aggregation. Due to the lower affinity of mutant SOD1 for superoxide, the formation of peroxynitrile molecules and other RNS is significantly increased, with the end result of RNS and ROS-mediated apoptosis [144]. Furthermore, peroxynitrile and RNS have been proposed as a cause for SOD1 aggregation and dysfunction themselves, through SNOs, mainly PDI S-Nitrosylation [145], as well as TAR DNA-binding protein 43 (TDP43) [138]. 

RNA binding dysregulation can occur either because of misfolding and aggregation of TDP43, or because of mutant FUused in Sarcoma/Translocated in LipoSarcoma (FUS). Intracytoplasmic aggregation of nuclear TDP43 is the most common finding in ALS patients, however, mutations of the TARDBP gene, which encodes TDP43, are an extremely rare cause of ALS [142]. Due to the intracytoplasmic aggregation, the nuclear levels of TDP43 and FUS decrease, leading to a deficit of RNA binding and abnormal transcripts processing [138,141,142].

Aggregation of misfolded TDP43 and cell-to-cell propagation are pathognomonic for a majority of cases of ALS. S-Nitrosylation, by facilitating the formation of a disulfide bond at critical cysteine residues, promotes the formation of abnormal TDP43 protein aggregates [146]. After aggregate formation, TDP43 proteinopathy can spread form cell to cell in a prion-like manner, contributing to the propagation of neuronal lesions [146,147,148,149]. Even more, TDP43 aggregate may trigger the misfolding and aggregation of wild-type SOD1 in both fALS and sALS cases [138], leading to the aforementioned RNS- and ROS-mediated apoptosis. This cross-protein misfolding interaction between TDP43 and SOD1 could explain the wild-type aggregation found in non-mutant SOD1 sALS patients reported by Forsberg et al [139]. Due to the positive feedback loop created by SOD1 dysfunction and increased generation of RNSs, mainly peroxynitrile, the antioxidant abilities of neurons fail and nitrosative stress levels rise. Through the nitration of tyrosine found in neurofilaments (NF), followed by NF ag-aggregation in motor neurons, Chou et al [143] link mutant SOD1 activity to the preferential death of upper and lower motor neurons characteristic of ALS.

**Figure 4:**
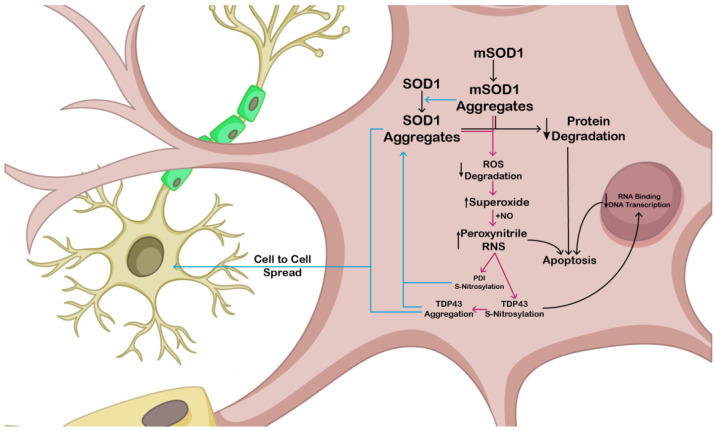
Increase in oxidative and nitrosative stress (red pathway), either through the increase formation of superoxide, decrease in ROS degradation, or increased NO concentration, represents the early stage. This increase culminates in the formation of peroxynitrile, and the S-Nitrosylation of key cellular proteins, such as PDI and TDP43, and the beginning of the prion-like stage (blue pathway). S-nitrosylated PDI and TDP43 are able to misfold wild-type SOD, causing a decrease in the ROS degradation capabilities of the neuron, further aggravating the early stage changes. Additionally, misfolded SOD and S-nitrosylated TDP43 are able to induce wild-type SOD misfolding in neighboring neurons. Finally, in the end stage (black pathway), the excessive protein accumulation, decreased DNA transcription, and direct toxicity of peroxynitrile, apoptosis occurs. Abbreviations: mSOD1, mutant copper-zinc superoxide dismutase 1; NO, nitric oxide; PDI, protein disulfide isomerase; RNS, reactive nitrogen species; ROS, reactive oxygen species; SOD1, wild-type copper-zinc superoxide dismutase 1; TDP43, TAR DNA-binding protein 43; ↑ increase; ↓ decrease. References: [15,135,137,138,140,141,143,144,145,146,147,148].

In the following graphic (Figure 4), we have summarized the main pathophysiological mechanisms in which NO is involved.

Finally, NO can serve as a link between the inflammatory changes associated with ALS and neuronal death, both as a trigger in the form of peroxynitrile and other RNS, as well as a mediator through the abovementioned pathways [150,151,152,153,154]. The major findings in this field have been summarized in the table below (Table 2).

## 5. Perspectives

There are important hopes regarding new therapies for neurodegenerative disorders that could influence the NO/NOS balance. The main targets of these therapies are to preserve as much neurons as possible, according to specific mechanisms associated with neurodegenerative diseases pathogenesis. There are many factors that are involved in neuronal death in PD, AD, or ALS, but certainly the NO/NOS system has an important role.

Since excessive NO production is detrimental despite its important physiological roles in lower concentrations, the key importance in limiting neuronal death and neurodegeneration progress seems to be a controlled reduction in NO synthesis, not a complete inhibition. Attempts finding an efficient therapy have been made by employing a variety of synthetic compounds, such as amino-acid amidine derivatives, urea, thiourea, other urea derivatives, aromatic amidines, and cyclic amidines, [155,156,157,158], as well as natural compounds or nutraceutical compounds [159,160,161].

Due to the complex involvement of NO in neurodegenerative disorders, with not only iNOS but also eNOS and nNOS being involved, less specific compounds, such as N5-(1-iminoethyl)-L-ornithine or 4-Methylaminopyridine [155,162,163], may offer a greater benefit in preventing and treating neurodegenerative conditions. However, research data are currently insufficient.

Factors such as adequate NO/NOS balance or BBB integrity have to be taken in account in each therapeutic strategy evaluation. A major issue in this research field is that promising results in animal studies have not translated to humans due to the lack of clinical trials and the lack of NOS inhibitors approved for human use [155]. Meanwhile, other compounds targeting different pathways are being tested in clinical trials [164].

Therefore, the research field in NO/NOS system contribution in neurodegenerative diseases’ pathogenesis has more to decipher yet, and clinical experimental studies are the first that should aid this objective.

## Figures and Tables

**Table 1:** Interplay between NO and dopamine levels.

Causal Trigger	Effect
S-Nitrosylation of tyrosine hydroxylase	↑ Dopamine synthesis Indirect increase in dopamine metabolites
Dopamine activation of mtNOS	↑ NO synthesis
↑ Peroxynitrile	↓ Dopamine re-uptake
↑ Glutamate	↑ NO synthesis ↑ Dopamine oxidation
↑ NO ↑ Peroxynitrile	↑ Dopamine oxidation

Abbreviations: mtNOS, mitochondrial nitric oxide synthase; NO, nitric oxide; ↑ increase; ↓ decrease. References: [7,67,81].

**Table 2:** Major findings regarding inflammatory responses in ALS.

Trigger Factor	Effect	Result	Ref.
LPS	Activation of mSOD1 bearing astrocytes	↑ iNOS ↑ NO	[150]
Mutant SOD1	Activation of astrocytes and microglial cells	↑ iNOS ↑ NO	[151]
Peroxynitrile or LPS	Activation of astrocytes	↑ iNOS ↑ NO Cytotoxic phenotype transformation	[152]
Peroxynitrile	Activation of astrocytes and microglial cells	↓ Astrocytic glutamate uptake ↑ Neuronal excitotoxicity	[153]
Peroxynitrile or LPS	Activation of astrocytes	↑ iNOS ↑ NO ↑ nerve growth factor ↑ nitrotyrosine ↑ death of p75(NTR)+ neurons	[154]

Abbreviations: iNOS, inducible nitric oxide synthase; LPS, lipopolysaccharide stimulation; nSOD1, mutant copper-zinc superoxide dismutase 1; ↑ increase; ↓ decrease.

## Data Availability

No new data were created or analyzed in this study.
